# Conducting polymer-inorganic nanocomposite-based gas sensors: a review

**DOI:** 10.1080/14686996.2020.1820845

**Published:** 2021-01-06

**Authors:** Yan Yan, Guiqin Yang, Jian-Long Xu, Meng Zhang, Chi-Ching Kuo, Sui-Dong Wang

**Affiliations:** a College of Electronic and Information Engineering, Shenzhen University, Shenzhen, P. R.China; b School of Physics and Electronic Engineering, Yuxi Normal University, Yuxi, Yunnan, P. R. China; c Institute of Functional Nano & Soft Materials (FUNSOM), Jiangsu Key Laboratory for Carbon-Based Functional Materials & Devices Soochow University, Suzhou, Jiangsu, P. R. China; d Institute of Microscale Optoelectronics (IMO), Shenzhen University, Shenzhen, P. R. China; e Institute of Organic and Polymeric Materials, Research and Development Center of Smart Textile Technology, National Taipei University of Technology, Taipei, Taiwan

**Keywords:** Polymer, gas sensors, polymer-inorganic nanocomposites, nanostructure, synergistic effect, 103 Composites, 201 Electronics / Semiconductor / TCOs, 208 Sensors and actuators

## Abstract

With the rapid development of conductive polymers, they have shown great potential in room-temperature chemical gas detection, as their electrical conductivity can be changed upon exposure to oxidative or reductive gas molecules at room temperature. However, due to their relatively low conductivity and high affinity toward volatile organic compounds and water molecules, they always exhibit low sensitivity, poor stability, and gas selectivity, which hinder their practical gas sensor applications. In addition, inorganic sensitive materials show totally different advantages in gas sensors, such as high sensitivity, fast response to low concentration analytes, high surface area, and versatile surface chemistry, which could complement the conducting polymers in terms of the sensing characteristics. It seems to be a win-win choice to combine inorganic sensitive materials with polymers for gas detection due to their synergistic effects, which has attracted extensive interests in gas-sensing applications. In this review, we summarize the recent development in polymer-inorganic nanocomposite based gas sensors. The roles of inorganic nanomaterials in improving the gas-sensing performances of conducting polymers are introduced and the progress of conducting polymer-inorganic nanocomposites including metal oxides, metal, carbon (carbon nanotube, graphene), and ternary composites are presented. Finally, a conclusion and a perspective in the field of gas sensors incorporating conducting polymer-inorganic nanocomposite are summarized.

## Graphical Abstract

**Figure:**
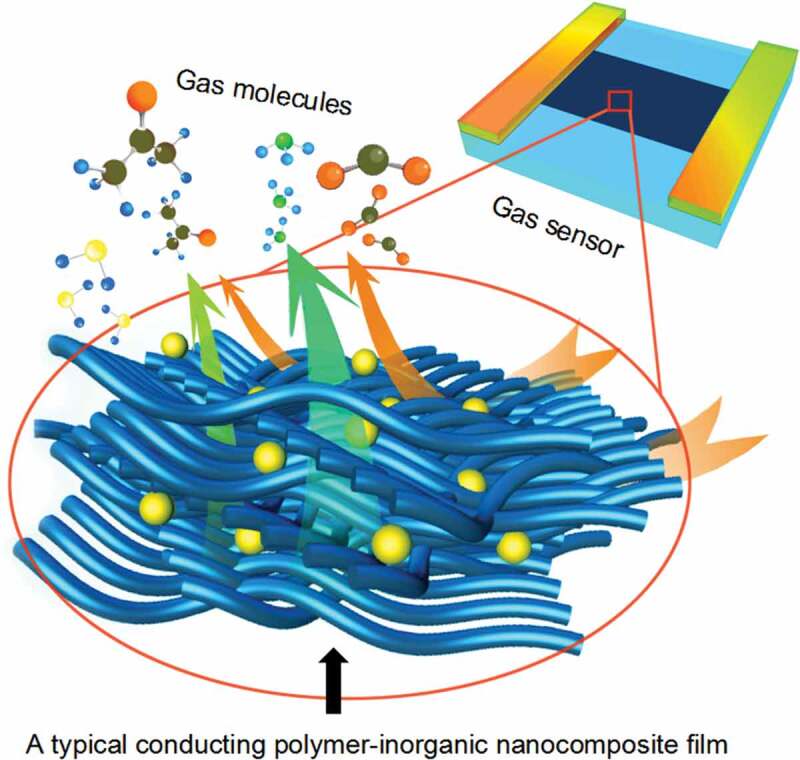

## 1. Introduction

**Figure 1.**
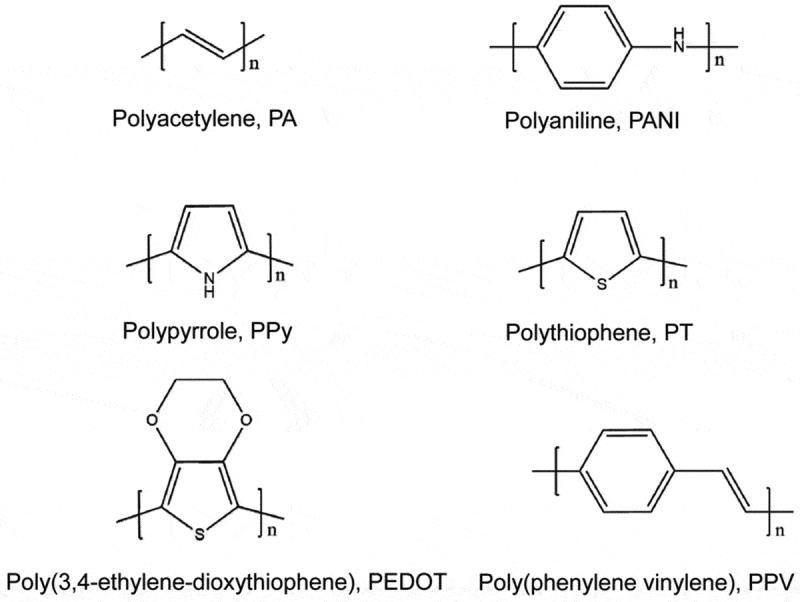
Chemical structures of representative conducting polymers

Conducting polymers have undergone rapid development and shown great potential in room-temperature chemical gas sensors since their electrical conductivity can be changed upon exposure to oxidative or reductive gas molecules at room temperature [1,2]. In general, the conducting polymers with typical π-conjugated structures show p-type conductive behaviors. Upon interaction with gas molecules, they behave either as an electron donor or an electron acceptor, which can result in the increase or decrease of carrier concentration, hence the change in electrical conductivity or resistance of the sensing polymers. Conducting polymers reported as sensitive materials in the literature mainly include polyacetylene (PA), polyaniline (PANI), polypyrrole (PPy), polythiophene (PT), poly(3,4-ethylenedioxythiophene) (PEDOT), poly(phenylene vinylene) (PPV) and their derivatives (Figure 1). In the undoped state, conducting polymers are either electrical insulators or semiconductors. To increase their conductivity, a doping process such as protonic acid doping or redox doping is applied to polymers, which is accompanied by removing the electrons on the backbones. Positive charges remained in the backbone act as the charge carriers, which could increase the conductivity from the low level of an insulator or semiconductor (10^−10^-10^−5^ S/cm) to the conducting level (1–10^5^ S/cm). The unique tunable electrical properties, together with easy synthesis, structural diversity, facial functionalization, and flexibility of these conducting polymer materials enable diverse energy and electrical device applications [3]. Particularly, this unique doping/dedoping process allows conducting polymers to be explored as promising candidates for room-temperature gas sensor applications.

With significant efforts in the past decades, there has been a great research progress in conducting polymers based room-temperature gas sensors. However, due to their relatively low conductivity and high affinity toward volatile organic compounds (VOCs) and water molecules, they always exhibit low sensitivity, poor stability, and gas selectivity, which hinder their practical gas sensor applications. Many efforts have been made for sensing performance enhancement including increasing the active surface area, redox doping, functionalization, etc. For example, Huang et al. [4] demonstrated a highly porous sensing layer composed of one-dimensional (1D) PANI nanofibers with superior sensing performances including high sensitivity, selectivity, and rapid response owing to its higher surface area compared with bulk films. Such gas-sensing enhancement effects arising from the low-dimensional structures are also reported in other conducting polymers including PPy, PT, etc. Nevertheless, although the sensing responses are greatly improved, the gas-sensing behaviors are not highly reversible and reproducible, which is a main concern for practical sensor applications. Zhang et al. [5] demonstrated (+)-camphor-10-sulfonic acid (HCSA) doped polyaniline nanofibers by electrospinning methods, which exhibited greatly improved response/recovery behaviors to 500 ppm NH_3_. Moreover, Kwon et al. [6] demonstrated that PPy functionalized with the carboxyl groups (-COOH) showed specific selectivity to dimethyl methyl phosphonate (DMMP) gas due to the intermolecular interactions between -COOH groups and phosphoryl groups of DMMP molecules. However, although the gas sensing performances were improved, their limitations such as low sensitivity, reversibility, and selectivity remained a challenge for practical use in gas sensors.

In contrast to conducting polymers, inorganic sensitive materials show totally different advantages in gas sensors, i. e. metal oxides always show high-sensitivity due to oxygen stoichiometry and active surface charge [7]; however, the requirement of high temperature for operation always hinders its wide applications; metal nanostructures are adopted as sensitivity promoters by chemical and electronic sensitization effects [8]; One-dimensional (1D) or two-dimensional (2D) carbon materials exhibit a fast response to low concentration analytes at room temperature owing to their low electronic noise, high surface area, and versatile surface chemistry, which could complement the conducting polymers in terms of the sensing characteristics [9,10]. The use of conducting polymer-inorganic nanocomposite may result in high-performance gas sensors due to their synergistic effects, which has attracted extensive interest in gas-sensing applications. In the nanocomposite system, the host organic and guest inorganic phases are interacted by weak van der Waals or hydrogen bonding or covalent or ionic covalent bonding, which could provide enhanced or novel chemical and physical functionalities. Such synergetic/complementary effects in the nanocomposite could help to eliminate their inherent drawbacks and also utilize the advantages of their individual constituents in gas-sensing fields, which could result in high-performance sensitive materials and gas sensors. In this review, we will summarize the recent development in polymer-inorganic nanocomposite toward high-performance gas sensors. In section 2, we first introduce the roles of inorganic nanomaterials in improving the gas-sensing performances of conducting polymers. In section 3, we mainly describe the progress of conducting polymer-inorganic nanocomposites including metal oxides, metal, carbon (carbon nanotube, graphene), and ternary composites, respectively. Finally, we give a conclusion and a perspective in the field of gas sensors incorporating conducting polymer-inorganic nanocomposites.

## 2. Comprehensive roles of inorganic nanomaterials in gas-sensing enhancement effects

### 2.1. Constructing P-N or Schottky heterojunctions

The gas-sensing responses of the conducting polymer-inorganic nanocomposite-based sensors are obtained by recording the time-dependent film resistance change as a function of target gas concentration. The film resistance value changes upon the exposure of reducing/oxidizing gas, and gradually restores to the original state when the target gas flow is removed. The gas-sensing response arises from the physical absorption of target analyte molecules onto the sensing films and the electron capture/donation process of the polymer matrix embedded with the inorganic nanomaterials, which is enhanced by the junction effects at the conducting polymer-inorganic interfaces.

When the conducting polymer matrix is embedded with inorganic nanomaterials, a P-N or Schottky heterojunction is formed at the conducting polymer/inorganic interfaces, depending upon the nature of inorganic nanomaterials [11–16]. Taking it for example, when n-type metal oxide nanostructures are introduced into the polymer matrix, a P-N heterojunction is formed at the polymer-metal oxide interfaces, accompanying with the formation of the depletion region in both polymer and metal oxides [16]. The interaction of the target gas molecules and the nanocomposite film surface lead to the decrease/increase of electrons in the polymers and therefore the change in the width of the depletion region, which could narrow/widen the conductive pathway of the polymers. Considering the concentration of doped metal oxide nanostructure to conducting polymers is low, usually in the order of 0.1–10 wt.%, the nanocomposite film resistance is mainly controlled by the conducting polymers. The synergistic effects of the changed conductivity and conductive pathway of polymers in the nanocomposite films result in the enhanced sensitivity of the nanocomposite films toward the target analyte.

### 2.2. Modulating film morphology

In addition to the interfacial interactions, efficient gas molecule absorption is also an important aspect to achieve a high sensing response because the physical absorption of gas molecules onto the film is the first step for gas detection. A highly porous layer with a large surface area, high pore volume, and desired pore size could introduce more active sites and increase the gas molecule absorption. By introducing inorganic nanomaterials, the film morphology of the nanocomposite films and the gas-sensing performances can be adjusted freely [13,15–20]. The inorganic nanomaterials could be a template for the polymerization of conducting polymers, where the nanocomposite morphology could be determined by the inorganic nanomaterials and polymerization methods. Therefore, with a nanocomposite development effort, an efficient methodology for performance enhancement could be employed to design new nanocomposite material systems and develop new synthetic strategies to well control the morphology of the nanocomposite films.

### 2.3. Improving electrical conductivity

The electrical conductivity of the sensitive films is also important for achieving a high sensing response. For achieving a high sensing response, charge carriers generated upon absorption of gas molecules should migrate easily and be collected at the electrode. Due to the low conductivity of organic materials, few charge carriers reach the electrodes, which results in poor sensing response. The doping methods have been reported to improve the electrical conductivity of conducting polymers. Particularly, a nanocomposite of conducting polymers and conducting inorganic nanomaterials is a simple and effective technique to improve the intra- and inter-chain mobility of charge carriers in the polymer chains [21–24]. Proper selection of inorganic nanomaterials can modify the electrical conductivity to the desired level for high sensing response. The inorganic nanomaterials with high electrical conductivity could compensate for the low conductivity of conducting polymers to prevent loss of electrical signals, thus obtaining a large sensing response. For example, Shirsat et al. decorated PANI nanowires with Au nanoparticles (NPs) (~70-120 nm) to achieve enhanced gas-sensing behaviors [24]. The PANI-Au nanocomposite chemiresistive sensor exhibited a significantly enhanced detection limit with good selectivity and reproducibility, which has been ascribed to the reaction between H_2_S molecules and Au and enhanced conductivity of PANI induced by the electron transfer from PANI (donor) to Au (acceptor).

## 3. Conducting polymer-based nanocomposite gas sensors

### 3.1. Metal oxide-conducting polymer nanocomposites

Conducting polymers are widely used as effective species for gas detection due to their excellent electrochemical and electronic properties, as well as the advantages of low cost, long-term stability, and easy synthesis. However, the disadvantages of their performance such as low sensitivity, slow response, and recovery process, poor thermal stability, and selectivity also limit their further applications in gas sensors. Fortunately, scientists have verified that polymer/metal oxide nanocomposites can not only reduce the defects of polymer or metal oxide but also effectively improve their sensitivity, thermal stability, and response speed. Through in-depth scientific research, people have a higher understanding of the mechanism responsible for the gas sensing performance enhancements. It is believed that the change of micromorphology and the formation of P-N junctions can effectively promote their sensing process.

With the attempt of different kinds of preparation methods, a variety of conducting polymer/metal oxide nanocomposites with form of thin films [25], particles [26–29], fibers [30–33], irregular shapes [34], sheets [35], short rods [36], tubes [37], flowers [38], networks [39–41] and core@shells [42,43] have been synthesized and applied to gas sensors successively.

**Figure 2.**
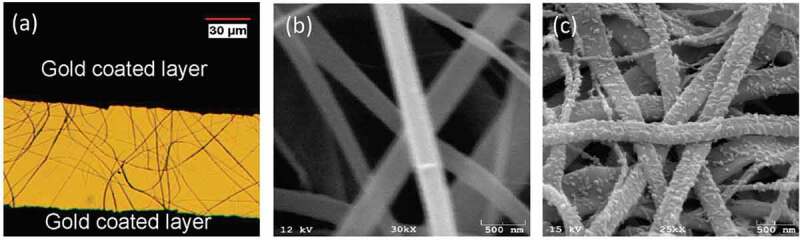
(a) An optical image of PANI/TiO_2_ nanocomposite-based sensor, (b) SEM images of TiO_2_ microfibers and (c) PANI/TiO_2_ nanocomposites. Reprinted with permission from [30]. Copyright 2020 American Chemical Society

**Figure 3.**
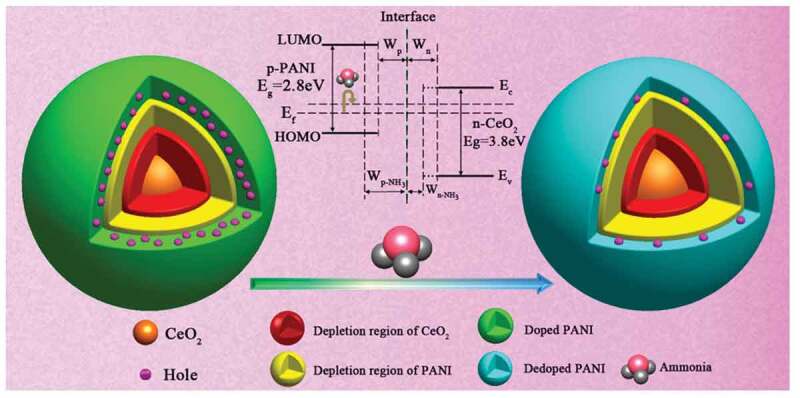
Schematic diagram about synergic effect of CeO_2_@PANI under NH_3_ gas. The inset was schematic diagram of P − N junction in equilibrium state. Reprinted with permission from [28]. Copyright 2014 American Chemical Society

Taking PANI-based nanocomposites for example, as early as 2007, Jiang et al. [25] successfully prepared gas sensors for NH_3_ detection using hybrid PANI/TiO_2_ nanocomposites. They observed that compared with mono-phase PANI-based sensors, hybrid nanocomposites based sensors showed higher sensitivity, quicker responsivity, better stability, and shorter recovery time. After that, different kinds of nanostructured metal oxides including SnO_2_ [44–48], TiO_2_ [30], Fe_2_O_3_ [32,49], GeO_2_ [28,50], ZnO [34,51,52], WO_3_ [38,39,41,43], Nb_2_O_5_ [53] and MoO_3_ [36] are tried to be combined with PANI for gas detection. In 2010, sensors based on PANI/TiO_2_ nanofiber structures were firstly reported by Gong et al. [30] for dilute NH_3_ detection (Figure 2). They obtained the Ti^4+^ containing microfiber precursor by electrospinning method and then calcined the precursor at 600°C for 4 h to prepare TiO_2_ microfibers. After that, the P-N heterojunction nanohybrids with PANI nano-grain enchased TiO_2_ microfibers were formed through a polymerized reaction. The PANI/TiO_2_ based sensors showed high sensitivity to 50 ppt of NH_3_. In the nanocomposites, the PANI NPs embedded on the surface of TiO_2_ microfibers acted as nano-switches. When the NH_3_ molecules were adsorbed onto the PANI/TiO_2_ nanocomposites, the PANI NPs can turn off the current loop, and when the gas molecules were desorbed, the circuit was reconnected. The NH_3_ gas sensitivity was significantly improved with the rapid increase of sensor resistance. The next year, Wang et al. [28] fabricated NH_3_ gas sensors using core-shell CeO_2_@PANI structure. The sensors exhibited a high response of 6.5 to 50 ppm under NH_3_ detection and great long-term stability. The internal mechanism of device performance improvement was analyzed in detail. They demonstrated that the increased sensitivity and stability benefitted from the P-N heterojunction of nanohybrids. The electron-donating NH_3_ changed the original space charge region at the equilibrium condition, which decreased the hole concentration in the PANI, and enlarged the depletion region from W_p_ to W_p-NH3_. The conduction paths were thus reduced from the thicker emeraldine salt (ES) formed shell painted in green to the thinner emeraldine base (EB) formed shell painted in blue. Since the inherent resistance of CeO_2_ was extremely high, the entire resistance of the hybrid system increased finally (Figure 3).

In recent years, the fabrication of electronic devices based on organic/inorganic hybrid systems on flexible/ductile substrates has become a new research hotspot. Compared with the traditional electronics, flexible electronics can adapt to different working environments to a certain extent, and meet the deformation requirements of the equipment.

**Figure 4.**
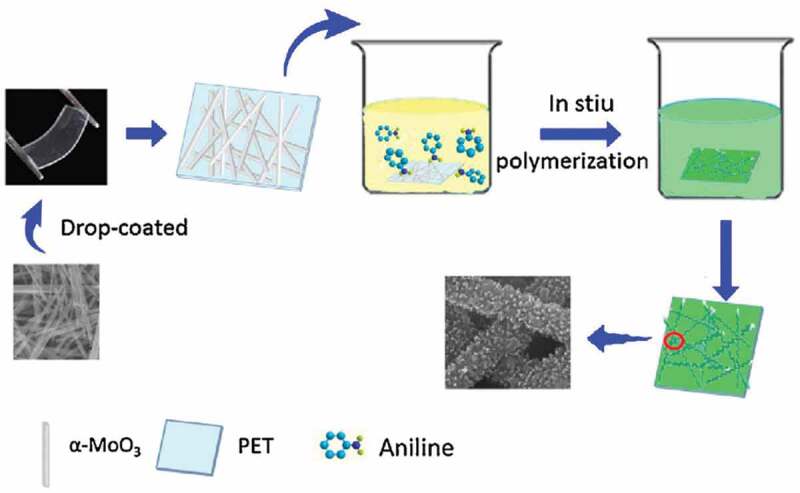
Schematic diagram of fabrication process of α-MoO_3_/PANI nanocomposites. Reprinted with permission from [36]. Copyright 2017 Elsevier

**Figure 5.**
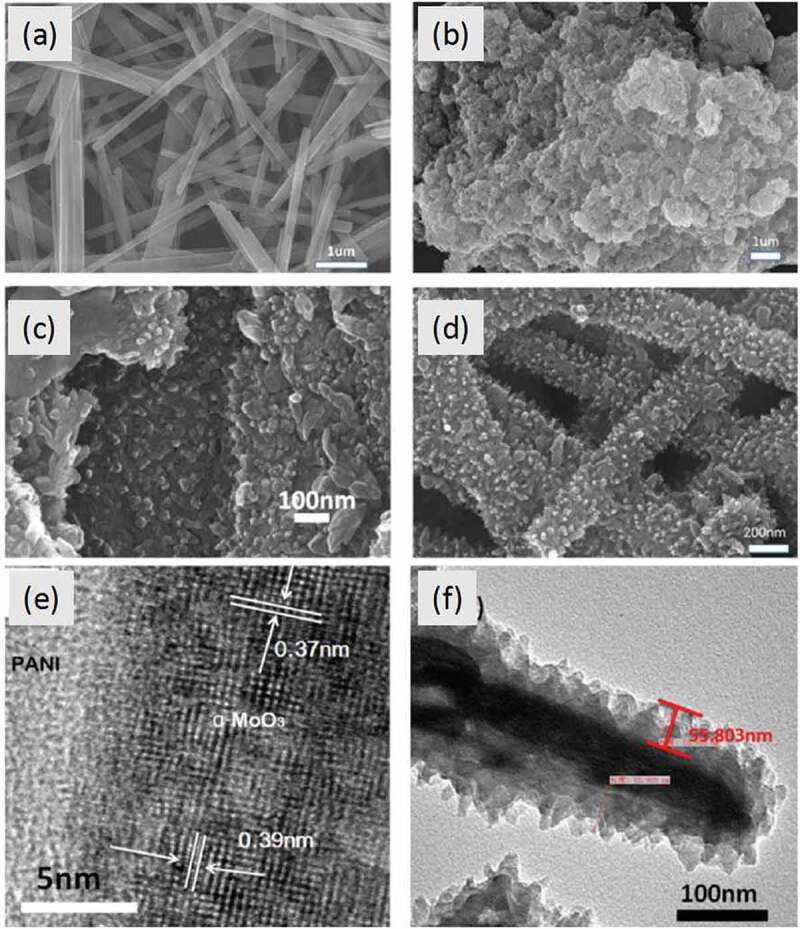
SEM images of (a) MoO_3_ nanorods, (b) PANI powder, (c) and (d) MoO_3_/PANI films with different contents of MoO_3_ (e.g. 0.1 mg, 0.3 mg, and 1 mg); (e, f) TEM images of the α-MoO_3_/PANI nanocomposites.Reprinted with permission from [36]. Copyright 2017 Elsevier

In 2017, Bai et al. [36] reported nanorod-like α-MoO_3_/PANI-based triethylamine (TEA) sensors. The sensors were fabricated on a flexible polyethylene terephthalate (PET) substrate with the PANI film covered onto the MoO_3_ nanorod framework (Figure 4). In this work, comparative experiments were carried out to optimize the quality of the hybrid system and related device performance (Figure 5). The optimized sensors demonstrated high selectivity and good sensitivity of 5.5 to 10 ppm TEA at room temperature. They considered that the improved response after adding MoO_3_ nanorods into PANI was possibly caused by two factors. On one hand, the PANI embedded on the surface areas of MoO_3_ robs formed a network that provided a large number of α-MoO_3_/PANI interfaces, which enhanced the adsorption efficiency of gas molecules. In addition, the new formed structure was conducive to the diffusion of gas molecules in the nanocomposite system. On the other hand, the more important factor for enhancing the sensing response was new-formed P-N heterojunctions, which reduced the enthalpy and activation energy of physical adsorption of gas molecules, leading to good electron-donating characteristics.

It is worth mentioning that gas sensors based on hybrid PANI/WO_3_ nanocomposites on flexible PET substrates were also reported by Li et al. Flower-like [38] and hollow spheres [43] WO_3_@PANI nanocomposites were designed and applied in sensors for room-temperature NH_3_ detection. The highest response value was 20.1 for flower-like WO_3_@PANI and 25 for hollow sphere WO_3_@PANI to 100 ppm NH_3_ at room temperature.

Besides PANI, PPy is also one of the most ideal sensing candidates in the VOCs detection, since it possesses obvious advantages of good environmental stability, low operating temperature, easy synthesis, and reversible redox reaction [26,54–56]. Generally speaking, similar to the preparation of PANI/metal oxide nanocomposites, the typical process of preparing conductive PPy/metal oxide nanocomposites usually includes the following three steps: first, nanostructured metal oxides were prepared by chemical bath deposition and in air calcination [57], in-situ growing [35], hydrothermal synthesis, sol-gel method [54], surfactant-assisted method, and calcination [33,40,55], template-based hydrothermal synthesis [42,56], ultrasound-assisted precipitation method [26] or carbon microspheres templated method [56,58]. Then, conducting polymer was synthesized by in-situ oxidative polymerization method [29], electrochemical deposition [57], chemical oxidation polymerization [26], or solution method [35,37,55]. Finally, metal oxides are combined with polymers through the interface reaction of inorganic and organic materials. In order to combine organic and inorganic materials closely to form effective PN junction and obtain high adsorption areas, a special treatment process is necessary.

**Figure 6.**
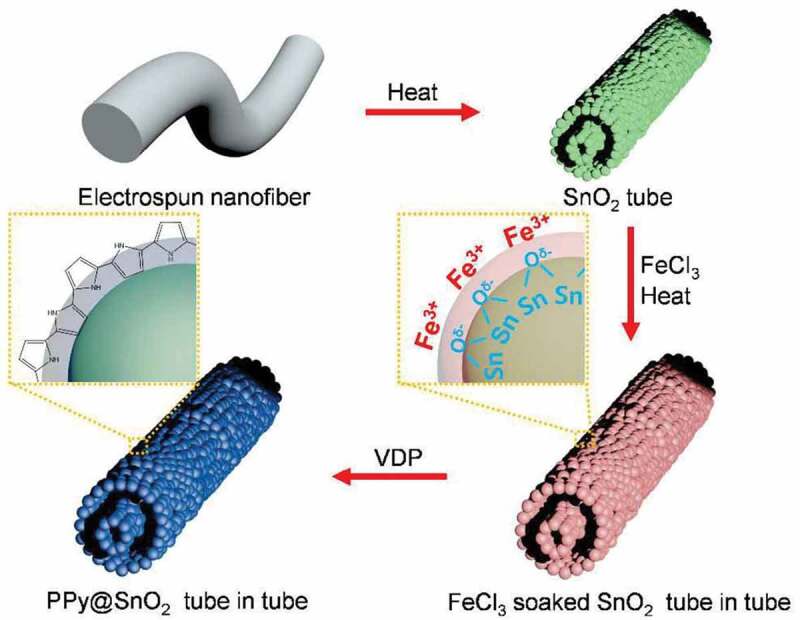
Schematic diagram of fabrication process of the SnO_2_@PPy tube-in-tube structure. Reprinted with permission from [37]. Copyright 2013 Royal Society of Chemistry

For instance, Jun et al. [40] prepared sensors based on tube-in-tube SnO_2_@PPy construction for detecting DMMP at extremely low concentrations (0.05 ppb). The tube-in-tube construction was fabricated via a single nozzle electrospinning method with two kinds of mixed solvents, i.e., N,N-dimethylformamide (DMF) and ethanol. By the control of calcination conditions and the successive evaporation of solvents with different boiling points, the tube-in-tube construction was fabricated. Then, the ferric cations (Fe^3+^) were modified onto the surface of SnO_2_, followed by a vapor deposition polymerization step in the vacuum environment to form the organic/inorganic hybrid structure (Figure 6). Dongzhi Zhang et al. [56] demonstrated NH_3_ gas sensors based on PPy/Zn_2_SnO_4_ nanocomposite with extremely high sensitivity. PPy nanospheres were combined with Zn_2_SnO_4_ hollow spheres through a layer-by-layer alternative deposition. It is worth mentioning that in addition to PANI/metal oxides and PPy/metal oxides based gas sensors, PT/metal oxides [58,59] and poly(3,4-ethylenedioxythiophene):poly(4-styrenesulfonic acid) (PEDOT: PSS)/metal oxide [60–62] based sensors have also been fabricated and studied.

In summary, there are two main advantages of doping metal oxide into conducting polymer to form the heterostructure. On one hand, the introduction of metal oxides can adjust the electrical properties of conducting polymers and form a unique P-N junction with chemical electron conduction property. On the other hand, nanostructured metal oxides with various morphologies can be easily introduced into conductive polymers, thus greatly increasing the surface areas of the mixture. In Table 1, the notable examples of the sensors based on the hybrid system of conducting polymers and metal oxides developed in the recent decade are listed, and their main sensing properties are summarized.

**Table 1.** Conducting polymer/metal oxides hybrid composites used in gas sensors

Polymer	Metal oxides	Target gas	Concentration (ppm)	Response	Response/recovery Time (s)	T (°C)	Ref.
PANI	TiO_2_	NH_3_	23	1.67	18 ~ 58	25	[25]
		CO	140	~1.9			[25]
		NH_3_	50 ppt	0.4%		RT	[30]
	SnO_2_	NH_3_	100		15 ~ 80		[44]
		NO_2_	50 ppb		5 ~ 15 (min)	25	[46]
		SO_2_	2				[46]
		NO_2_	37		17 ~ 25	140	[47]
		NH_3_	100	29		21	[45]
		NO_2_	10	~2			[45]
		NO_2_+ NH_3_	10	~5			[45]
		CO	25	15		30	[48]
	Fe_2_O_3_	LPG	50	0.5	< 60	28	[49]
		NH_3_	10.7	3070%		20	[32]
	GeO_2_	NH_3_	50	6.5		RT	[28]
		NH_3_	50	262.7%		25	[50]
		NO_2_	50	~40%			[50]
		HCHO	50	~20%			[50]
		H_2_S	50	~13%			[50]
		CO	50	~10%			[50]
		SO_2_	50	~5%			[50]
		O_2_	50	~5%			[50]
	ZnO	NH_3_	100	2.5		27	[34]
		NH_3_	20	14%	< 35	300 K	[51]
		NH_3_	10	2150%	431 ~ 387	RT	[52]
	Nb_2_O_5_	LPG	500	45.21%	30 ~ 50	RT	[53]
	WO_3_	NH_3_	5	24%	136 ~ 137	RT	[39]
		NH_3_	100	158%			[39]
		NO_2_	100	~40%			[39]
		H_2_S	100	~6%			[39]
		CH_3_OH	100	~3%			[39]
		C_2_H_5_OH	100	~1.5%			[39]
		NH_3_	10	7.1	13 ~ 49	RT	[38]
		NH_3_	100	25	136 ~ 130	20	[43]
		NH_3_	1	9%	< 32	RT	[41]
	MoO_3_	TEA	10	5.8		RT	[36]
PANI:PSS	Fe_2_O_3_	NO_2_	0.5	~8%		RT	[63]
PPy	SnO_2_	NH_3_	3		< 15	RT	[42]
		NH_3_	10.7	75%	259 ~ 468	RT	[31]
		DMMP	0.05 ppb	0.5%	1 ~ 30	RT	[37]
		NH_3_	0.1	57%	18 ~ 30	RT	[33]
	ZnO	LPG	1400	32.5%	4 ~ 40 min	RT	[26]
		NH_3_	0.5	21%	256 ~ 370	RT	[35]
		CH_3_NH_2_	280 μg	50%	< 50	100	[40]
	TiO_2_	LPG	1040	55%	112 ~ 131	RT	[57]
	FeOOH	DMMP	0.01	~7.5%	1.5 ~ 8.5	RT	[27]
	WO_3_	H_2_S	1	83%	6 ~ 210 min	RT	[64]
		NO_2_	100	61%		38	[54]
		TEA	100	680	7 ~ 49	RT	[29]
		C_3_H_6_O	0.37 μg		< 5	90	[55]
	Zn_2_SnO_4_	NH_3_	100	82.1%	35 ~ 26	RT	[56]
PPy:DBSA	WO_3_	NO_2_	100	72%	288 ~ 5990	38	[65]
PT	SnO_2_	NO_2_	100	3.69		90	[58]
	WO_3_	H_2_S	100	1.35	< 15	70	[59]
PEDOT:PSS	TiO_2_	NO_2_	0.005			RT	[61]
	WO_3_	NO_2_	0.05	~1.2	45.1 ~ 88.7	RT	[62]

### 3.2. Metal-conducting polymer nanocomposites

As a kind of catalyst with high electrocatalytic activity and good stability, metal nanostructures based on such as Pd, Pt, Au, have been widely studied in gas sensing. Relative to the precious metal NPs, metal nanostructures based on Ag and Cu are also commonly used high-efficiency catalysts with low-cost and can effectively improve the thermal stability, conductivity, and adsorption performance of gas sensors. Doping metal NPs into the polymer can significantly change the electrical properties of the newly formed system, and exhibit excellent adsorption and desorption capacity of reducing gases such as H_2_, CO, NH_3_ at room temperature. It is proved that the electrical conductance of the composites is sensitive to the shape, size, and content of metal nanophase. Various species of nanostructured metals including Pd [66], Au [67], Ag [68], Pt [69], and Cu [70] were used to combine with conducting polymers through different approaches such as hydrothermal synthesis [19,66,71], in-situ chemical oxidative polymerizing [68,70,72–76], self-assembling [67,69,77], electrodeposition [78], chloroaurate anion reduction [79], template-based vapour deposition polymerization (VDP) [80], and in-situ photo-polymerization [81,82]. At the same time, the combination of different kinds of polymers with specific NPs has also been widely studied. Take Ag NPs as an example, PANI/Ag [68,73,81,82], PPy/Ag [72,74], and PEDOT/Ag [80] composite systems has been fabricated and applied to gas sensors.

Early in 2005, Athawale et al. [66] reported a work about PANI/Pd nanocomposites synthesized by reflux method. They exposed PANI/Pd nanocomposites to different aliphatic alcohol vapor of methanol, ethanol, and isopropanol and found that the sensors based on PANI/Pd nanocomposites showed high sensitivity, well selectivity, and fast response towards methanol vapor. After then, the gas response of different kinds of metal/polymer nanocomposites has been widely studied. Among them, the influence of concentration, size, shape, and type of metal nanomaterials on device performance has become a research hotspot. Specifically, in 2009, nanocomposites of PPy/Pd were fabricated by Hong et al. [19] through solution reduction of Pd ions and subsequent gas-phase polymerization of pyrrole. They found that the concentration and ratio of reactants, polymerization reaction time, and the content of polymer additives would seriously affect the morphology of the composite membrane, and finally determined the sensitivity of the nanocomposite to NH_3_ detection. In this work, they demonstrated that the gas-sensing properties of nanocomposites were greatly affected by the size of Pd NPs and the configuration of composite membranes. With the addition of PVP, the size of NPs can be reduced to less than 10 nm and better distribution can be achieved. The gas sensors based on the composite of PPy and small-sized Pd showed a higher response than PPy alone because the well-distributed Pd NPs provided more active surface reaction sites and affected the charge transfer between polymer and reaction gas molecules.

**Figure 7.**
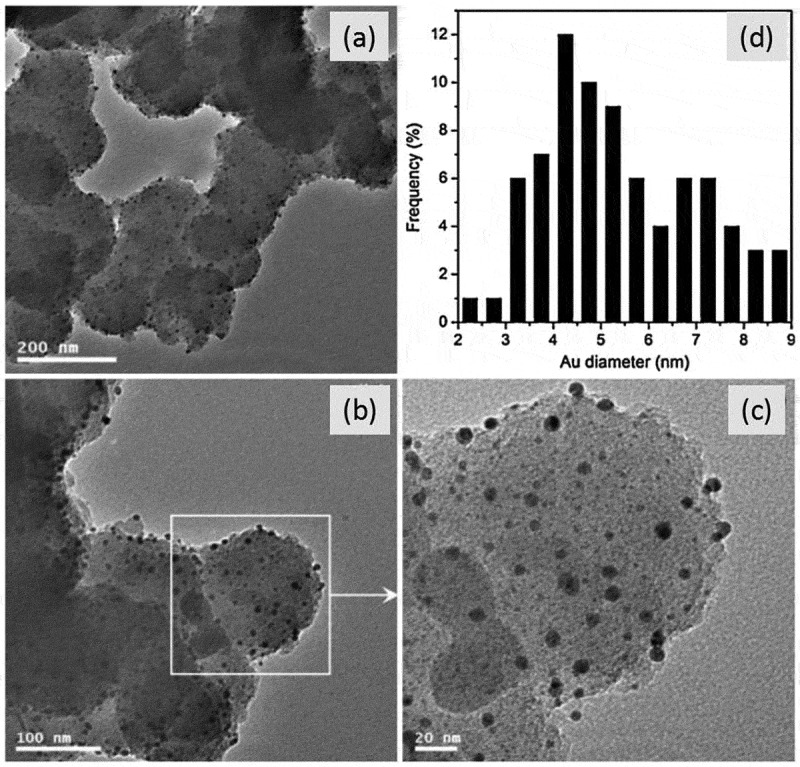
(a–c) TEM images of the hybrid PPy/Au system and (d) size distribution of Au NPs. Reprinted with permission from [75]. Copyright 2013 Elsevier

**Figure 8.**
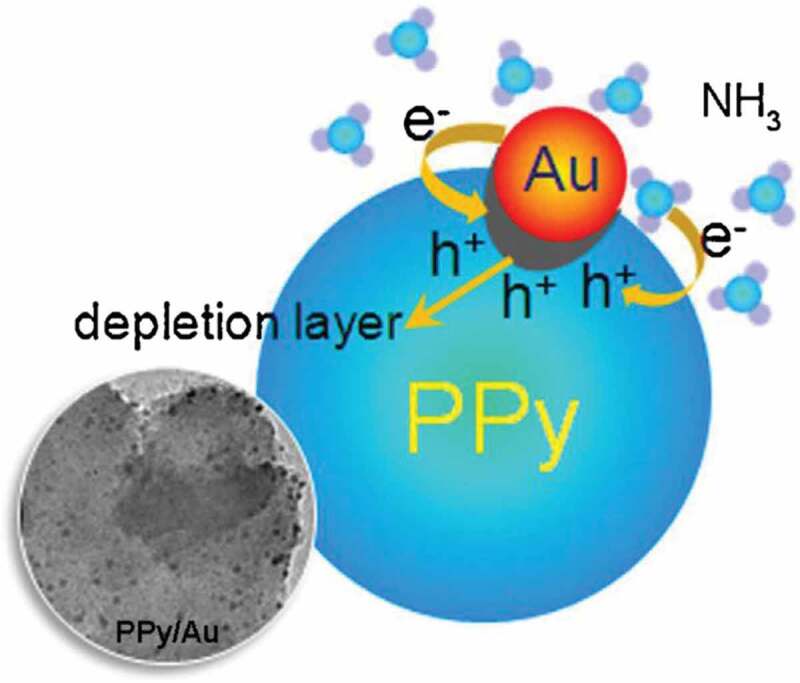
Possible gas detection mechanism of PPy/Au hybrids. Reprinted with permission from [75]. Copyright 2013 Elsevier

In 2013, Zhang et al. [75] developed a one-pot strategy method to prepare PPy/Au nanocomposites with uniform Au NPs dispersed on PPy. By introducing material lysine, the size of Au NPs was significantly reduced. Meanwhile, there was a good consistency distribution for the NPs in the hybrid system (Figure 7). The Au NPs became very active when their sizes were smaller than 5 nm due to the quantum size effect, which is helpful for NH_3_ molecules to adsorb on the PPy sensing layer in the presence of atmospheric O_2_. The sensitivity of PPy/Au nanocomposites for room-temperature detection reached to about 1.47 for 300 ppm NH_3._ They contributed the obviously enhanced sensor properties to the uniform small-sized Au NPs distribution. The possible mechanism was also discussed (Figure 8). Conducting polymer PPy is a kind of p-type semiconductor. In the PPy/Au hybrid system, the introduction of Au NPs will form the ‘nano Schottky effect’ at the interface, and then form a depletion layer, thus increasing the resistance of the whole system. In the presence of electron-doped NH_3_, the holes in the hybrid system will be consumed and the resistance will be increased.

In addition to the size of NPs, the concentration of NPs is another important research point. In 2009, Arup Choudhury [68] synthesized PANI/Ag nanocomposites at the concentration of 0.5, 1.5, and 2.5 mol% Ag by in-situ chemical polymerization. It was found that the sensitivity and response/recovery time of PANI/Ag nanocomposite-based sensor was depended on the concentration of Ag NPs. Compared with pure PANI, the dielectric and conductivity of PANI/Ag nanocomposites were significantly improved, and the AC conductivity was increased by about 100 times. The sensors based on PANI/Ag nanocomposites showed faster and more reversible performance in ethanol detection. When the concentration of Ag was 2.5 mol%, the sensors showed the highest sensing capacity and the best long-term stability. After then, Park et al. [80] reported the study of NH_3_ chemical sensors based on Ag NPs/PEDOT nanotube composites. The concentration of Ag NPs was changed by controlling the AgNO_3_ with 5%, 10%, 30%, and 40% (w/w). The sample Ag NPs/PEDOT nanotube with 5 wt% exhibited the highest sensitivity and the lowest detection limit to NH_3_. However, it was worth noting that although the higher concentration of Ag NPs can increase the surface reaction sites and improve the conductivity when the concentration was too high, the Ag NPs would agglomerate, and destroy the original morphology, which played a negative role in the gas-sensing response.

Generally, in the metal-conducting polymer nanocomposite system, metals often exist in the form of NPs, while conductive polymers often exist in the framework of thin films. However, one dimensional or quasi one-dimensional nanostructured polymer materials can also take advantage of their high surface volume ratio to optimize the application of gas sensors. A composite with a hierarchical network structure was prepared for NH_3_ detection on the flexible PET substrate using Ag nanowires as a framework and combining with one-dimensional-nanostructured PANI [73]. Even if the concentration was as low as 5 ppm, the sensitivity was linear with the increase of NH_3_ concentration. Compared to the particle-like PANI films, hierarchical PANI/Ag nanocomposites based films exhibited higher selectivity to NH_3_. They believed that the significant increase in sensing performance was attributed to the confined nanostructures of quasi one dimensional in the network like films, the special acid-base reaction, and the enlarged specific surface areas of the layered network of PANI nanostructures.

To sum up, metal NPs can improve the sensing properties of conducting polymers, mainly due to the following reasons: firstly, the introduction of metal NPs changes the conductivity of polymers. Secondly, certain kinds of metal NPs show a chemical affinity for specific gas substances, and metal NPs as chemical receptors enhance the selectivity of sensors. Last, the effective surface area of nanocomposites interacting with target gas is increased by introducing nano metals into conductive polymers. Table 2 summarizes the examples of gas sensors based on polymer/metal hybrid composites developed in recent 10 years and lists their main sensing characteristics.

**Table 2.** Conducting polymer/metal nanostructures hybrid composites used in gas sensors

Metal	Polymer	Target gas	Concentration (ppm)	Response	Response/recovery Time (s)	T (°C)	Ref.
Pd	PANI	CH_3_OH	10		2 ~ 720		[66]
		NH_3_	500	21.9		RT	[83]
		NH_3_	100	~10			[83]
		H_2_	100	~6.5			[83]
		CO_2_	100	~1.7			[83]
		C_2_H_5_OH	100	~3.5			[83]
		LPG	100	~2.2			[83]
	PPy	NH_3_	50	13.9%	14 ~ 148	RT	[19]
	P(ANI-*co*-ASA):PSS	H_2_	5		90 ~ 40	RT	[71]
Pt	PPy	H_2_	1000		~330	RT	[69]
		LPG				170	[76]
Cu	PANI	NH_3_	50	86%	7 ~ 160	RT	[70]
Au	PANI	NH_3_	100	~3	5 ~ 7		[67]
		H_2_S	1				[79]
		CH_3_SH	1.5				[79]
	PPy	NH_3_	100	~1.35	20 ~ 40	RT	[75]
	PT	CH_3_NH_2_	1				[77]
Ag	PANI	C_2_H_6_O	500	~7.25	102 ~ 52	RT	[68]
		TEA	39	0.73	< 120		[81]
		NH_3_	10	~9.1		RT	[73]
		H_2_S	10	100%	~360		[82]
	PPy	NH_3_	10	0.54		RT	[72]
		NO_2_	100	68%	148 ~ 500	RT	[74]
	PEDOT	NH_3_	50	~25%	2 ~ 7	RT	[80]

### 3.3. Carbon nanotube/graphene-conducting polymer nanocomposites

#### 3.3.1. Carbon nanotube-conducting polymer nanocomposites

Carbon nanotubes composed entirely of carbon atoms are very suitable for chemical sensing because of their unique wildly adjustable conductivity, excellent mechanical properties, and high environmental stability *[84–89]*. However, in gas-sensing applications, the material shows poor sensitivity and selectivity, which usually needs surface modification. Therefore, it seems to be a win-win choice to combine carbon nanotubes with polymer gas-sensing materials, which usually exhibit relatively low chemical stability and conductivity but good sensitivity and selectivity.

**Figure 9.**
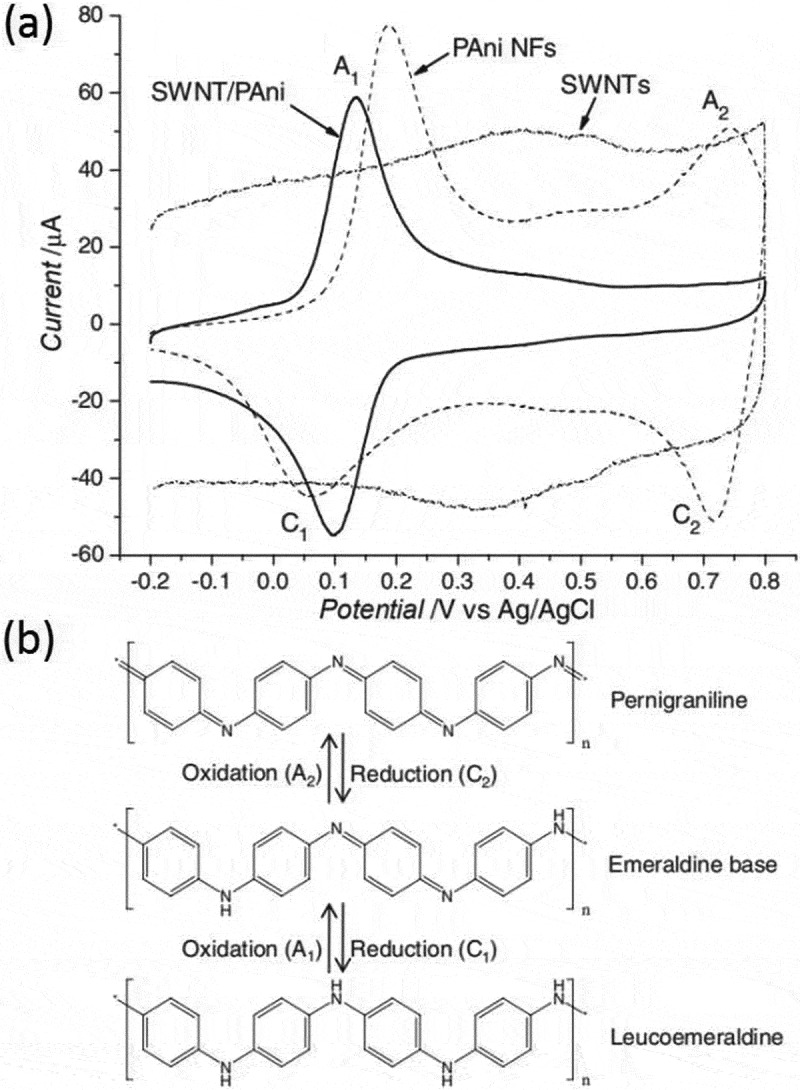
(a) Cyclic voltammograms of pristine SWNTs, PANI, and SWNT/PANI nanocomposites; (b) Three oxidation states of PANI. Reprinted with permission from [85]. Copyright 2010 John Wiley and Sons

In 2011, PANI/single-walled carbon nanotube (SWCNT) composites-based N_2_H_2_ sensors were fabricated by Ding et al. and the internal response mechanism was discussed *[85]*. They demonstrated that the electron transfer process was easier due to the effective interaction between the SWCNT core and the PANI shell (Figure 9). When exposed to the oxidizing atmosphere, electrons transferred from the SWCNT core to PANI shell, which effectively improved the oxidizing energy barrier between emeraldine/pernigraniline forms. When exposed to the reducing environment upon N_2_H_4_, the SWCNT core could promote the transition between emeraldine and leucoemeraldine formed of PANI, which made the sensing of PANI/CNTs composite reversible.

The combination of CNTs and polymer materials and the formation of good morphology and distribution are the key to affect the sensing performance. Therefore, the influence of the bonding approach on the quality and sensing properties of composites is also a challenging task. For instance, Liao et al. *[86]* used N-phenyl-p-phenylenediamine as an initiator to assist the polymerization process of PANI/SWCNT composite nanofibers. The nanocomposites showed widely tunable conductivities from 10^−4^ to 10^2^ S/cm. Chemosensors based on PANI/1.0 wt% SWCNT composite nanofibers showed a much more rapid response of 120 s to 100 ppb HCl and NH_3_ vapors compared to pure PANI nanofibers which response time was 1000 s. In 2017, tetra-b-carboxyphthalocyanine cobalt(II) (TcPcCo) was employed to fabricate ultra-fast PANI/multi-walled CNT (MWCNT) nanocomposites based NH_3_ gas sensor *[87]*. In this work, TcPcCo not only acted like a sensitive accelerator for gas sensors but also promoted the polymerization of MWCNTs and PANI to improve the conductivity of PANI in the form of dopant. Due to the synergistic effects of TcPcCo, PANI, and the MWCNTs, the sensors based on MWCNT/PANI hybrids showed an extremely rapid response/recovery time of 5.0/12.0 s to 100 ppm NH_3_. The highest sensitivity was 140.99% to 100 ppm NH_3_ and the lowest detection limit was 36 ppb.

**Figure 10.**
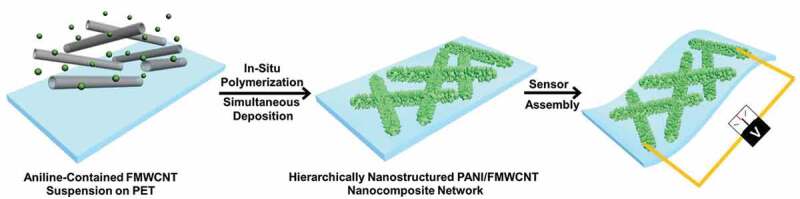
Schematic diagram of fabrication process of the hierarchically PANI/FMWCNT nanocomposites. Reprinted with permission from [88]. Copyright 2015 John Wiley and Sons

**Figure 11.**
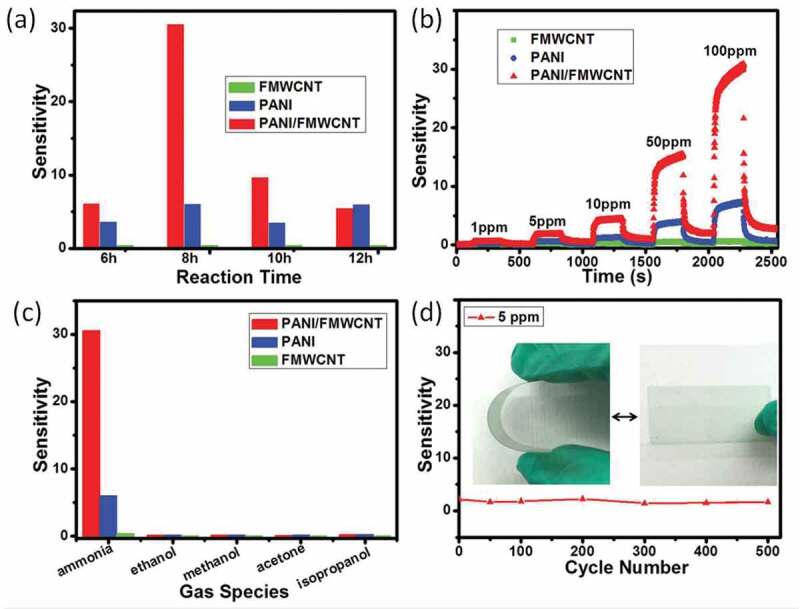
(a), (b) Gas-sensing sensitivity of sensors based on PANI/FMWCNT nanocomposite network; (c) selectivity and (d) flexibility of the sensors based on PANI/FMWCNT nanocomposite network. Reprinted with permission from [88]. Copyright 2015 John Wiley and Sons

Compared with polymer gas-sensing materials, CNTs exhibit another unique advantage of higher light transmittance. Therefore, it is also meaningful to fabricate gas sensors based on CNTs/polymer hybrid systems on flexible transparent substrates for some specific applications. Wan et al. *[88]* prepared a chemical gas sensor with transparent and flexible characteristics using a transparent functional multiwalled CNT (FMWCNT) network-based conductive film combined with nanostructured PANI nanorods. The nanocomposite thin film was fabricated through in-situ chemical-oxidizing polymerization method of aniline in a MWCNT liquid suspension (Figure 10). The flexible and transparent chemical gas sensor based on PANI/MWCNT nanocomposite film showed a high transmittance of 85% at 550 nm. Meanwhile, the sensor possessed excellent sensitivity at room temperature. After 500 bend/stretch cycles, no significant degradation was observed in performance, showing good flexibility and portable wearable properties (Figure 11). They believed that CNTs improved the efficiency of electron transport and aggregation. At the same time, the hierarchical structures of the composites increased the surface area to volume ratio, thereby improving sensing performance and making sensors more reliable.

#### 3.3.2. Graphene-conducting polymer nanocomposites

Graphene, well known as a two-dimensional sp^2^ bonded honeycomb carbon sheet with single atomic layer thickness, is widely applied in chemical sensing, field-effect transistors, transparent conductive electrodes due to its interesting quantum Hall effect, excellent mechanical strength, high thermal conductivity, and large specific surface area. Graphene sensors exhibit good sensitivity to H_2_, NO_2_, NH_3_, H_2_O, CO, and other gases, and has become one of the most popular candidate sensors in the field of gas sensing. The excellent gas sensitivity of graphene is mainly due to the following two aspects: firstly, the two-dimensional honeycomb structure of graphene exposes all carbon atoms to the environment, providing a large surface area for the reaction with gas molecules. Secondly, the high-quality graphene lattice and its two-dimensional properties make it has lower electrical noise and easier to shield charge fluctuations than a one-dimensional system. Therefore, scientists have made a lot of exploration on the preparation methods of polymer/graphene nanocomposites and the related gas-sensing properties.

For example, in early 2010, Al-Mashat et al. *[90]* reported gas sensors based on PANI/graphene nanocomposites via a chemical synthetic method for H_2_ detection for the first time. In this work, PANI nanofibers were formed on the surface of graphene by ultrasonic treatment of graphene in a mixed solution of aniline monomer and ammonium persulfate. They found that the PANI/graphene nanocomposite-based gas sensors possessed a sensitivity of 16.57% to 1% H_2_ gas, which was much higher than that of pure graphene sheets and PANI nanofibers. For NH_3_ detection, Wu et al. *[91]* fabricated PANI/graphene composites synthesized by chemical oxidative polymerization. The results indicated that PANI/graphene-based sensors exhibited a sensitivity of about 5 times higher than that of pure PANI, showing an approximately linear relationship within NH_3_ concentration ranging from 1 to 6400 ppm. The minimum detection limit of PANI/graphene sensors was about 1 ppm, approximately one-tenth of the detection limit of pure PANI. The preparation cost of graphene is very high, especially in large-area manufacturing. Therefore, reduced graphene oxide (rGO) with high surface area and relatively low cost becomes a good alternative material. Guo et al. *[92]* modified PANI NPs on rGO and finally integrated them into PANI nanofibers to form a hierarchical network film for NH_3_ detection. The optimized sensors showed a detectable concentration range from 100 ppb to 100 ppm, with a response time of 36 s and a recovery time of 18 s. Meanwhile, the network film exhibited an excellent transparency (90.3% at 550 nm) and reliable flexibility with no significant lack in performance after 1000 bend/extension cycles.

Generally speaking, nanostructured carbon possesses the advantages of a well-defined huge area to volume ratio and wide conductivity range. The carbon framework can be altered with functional groups, and the nanostructured carbon itself can also be modified on the conductive polymer as functional groups. Table 3 lists the notable examples of conducting polymer/CNTs and polymer/graphene nanocomposite-based sensors in recent 10 years and lists their main sensing performance.

**Table 3.** Conducting polymer/CNT and polymer/graphene nanostructures hybrid composites used in gas sensors

Doping material	Polymer	Target gas	Concentration (ppm)	Response	Response/recovery Time (s)	T (°C)	Ref.
SWCNT	PT	DMMP	25	~0.18		70	[84]
	PANI	N_2_H_4_	0.05				[85]
		NH_3_	0.1		120		[86]
CNT	PANI	NH_3_	300	2.07 MHz	375	45	[89]
MWCNT	PEDOT:PSS	NH_3_	5	~16%	900	RT	[93]
	PANI	NH_3_	100	30			[88]
		C_6_H_8_OS	100	~0.04			[88]
		CH_4_O	100	~0.03			[88]
		C_3_Cl_6_O	100	~0.02			[88]
		C_3_H_8_O	100	~0.04			[88]
		NH_3_	2	15.5%	6 ~ 35	RT	[94]
		CH_4_O	250	~3			[95]
		NH_3_	100	140.99%	5 ~ 12	RT	[87]
		NH_3_	25	46.9%		RT	[96]
Nitrogen-doped MWCNT	PPy	NO_2_	5	24.82%	~60	RT	[97]
Graphene	PANI	H_2_	1%	16.57%			[90]
		NH_3_	20	3.65	50 ~ 23	25	[91]
		NH_3_	20	~0.6		RT	[98]
	PANI:PSS	H_2_S	20	~60	90 ~ 150		[99]
GO	PPy	C_7_H_8_	200	~20			[100]
rGO	PANI	NH_3_	50	59.2%	3.4 ~ 10.4		[101]
		NH_3_	100	344.2	52 ~ 80	RT	[102]
		NH_3_	10	~6	36 ~ 18	RT	[92]
Nitrogen-doped graphene quantum dots (GQDs)	PANI	NH_3_	1500	110.92	540 ~ 588	RT	[103]
	PEDOT:PSS	NH_3_	1500	212.32%	462 ~ 600		[104]
		CO_2_	1500	~3%			[104]
		C_2_H_6_O	1500	~10%			[104]
		C_3_H_6_O	1500	~5%			[104]
		C_7_H_8_	1500	~9%			[104]
S and N co-doped GQDs	PANI	NH_3_	10	42%	115 ~ 44	RT	[105]

### 3.4. Polymer-based ternary nanocomposites

As mentioned above, the binary hybrid system composed of metal oxides, metal NPs, CNTs, or graphene with conductive polymers shows a good synergistic effect, which is conducive to optimizing the sensing characteristics. In order to further enhance the sensing performance, the research of ternary hybrid system has attracted more and more attention in recent years. Various gas sensors based on ternary nanocomposite have been synthesized recently for gas-sensing research, mainly include metal particles-metal oxide-conducting polymers [106–109], metal particles-carbon nanotubes-conducting polymers [110], metal particles-graphene-conducting polymers [111,112], metal oxide-graphene-conducting polymers [113–117], metal oxide-metal oxide-conducting polymers [118], metal oxide-metal oxide-metal oxide-conducting polymers [119].

**Figure 12.**
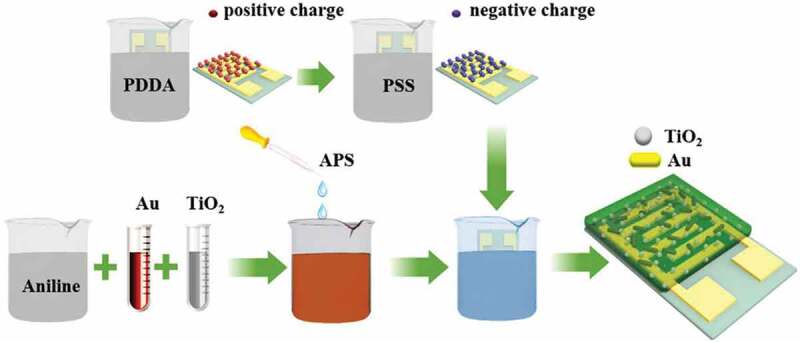
Schematic diagram of fabrication process of the PANI-TiO_2_-Au ternary nanocomposites. Reprinted with permission from [107]. Copyright 2017 Elsevier

In 2017, Liu et al. [107] applied an in-situ self-assembly method to fabricate Au-TiO_2_-PANI ternary nanocomposite thin film-based NH_3_ gas sensors (Figure 12). They tested response characteristics to NH_3_ concentrations ranging from 10 to 50 ppm at room temperature. The results showed that compared with the PANI-TiO_2_ binary film, the sensors based on PANI-TiO_2_-Au ternary composites performed a higher response value of 48.6% to 123% and shorter response time of 52 to 122 s, as well as better selectivity, and reversibility, which could be attributed to the synergy of nano junctions and the combined effect of Au nanorods catalysis.

**Figure 13.**
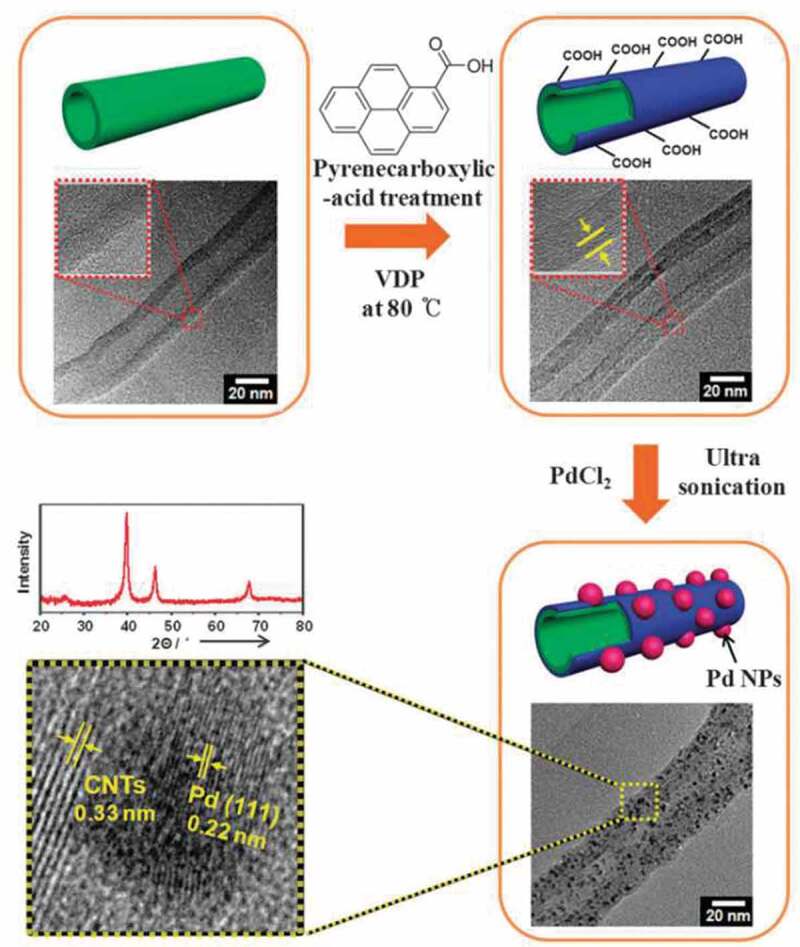
Fabrication process of the (CPPy)/CNTs/Pd nanocomposites. Reprinted with permission from [110]. Copyright 2015 Royal Society of Chemistry

Due to the success of CNTs and metal nanocomposites applied in gas sensing, researchers began to couple the metal NPs and CNTs with conductive polymers to obtain new composite sensing materials with better gas-sensing properties. For example, Park et al. [110] introduced a novel and simple method for preparing carboxylated polypyrrole (CPPy)/CNTs/Pd nanocomposites for NH_3_ detection (Figure 13). Through accurate material proportion and experimental condition optimization, the sensors possessed the minimum detection limit as low as 1 ppm to H_2_ and exhibited excellent reproducibility and reversibility.

In 2019, Zhang et al. [117] synthesized a kind of ZnO/graphene quantum dots (GQDs)/PANI nanocomposites through in-situ polymerization. When exposed to acetone atmosphere at room temperature, ZnO/GQDs/PANI nanocomposite sensors exhibited high sensitivity about 2% to 500 ppb acetone, short response/recovery time of 15/27 s, reliable repeatability, outstanding selectivity, as well as remarkable long-term stability. It should be noted that the ternary material mixture system based on the conductive polymer is not a simple random combination. In order to realize the synergistic reinforcement effect of various materials, process compatibility, morphology, and structure, composition ratio and function distribution should be considered comprehensively. Table 4 lists the notable examples of conducting ternary nanocomposite-based sensors developed in the recent decade and summarizes their main sensing performance.

**Table 4.** Conducting polymer/multicomponent nanostructures hybrid composites used in gas sensors

Polymer	Multicomponent		Target gas	Concentration (ppm)	Response	Response/recovery Time (s)	T (°C)	Ref.
PPy	Ag	SnO_2_	NH_3_	0.02	3.15%		RT	[106]
	Au	TiO_2_	NH_3_	0.02	3.2%		RT	[106]
	Pd	CNT	H_2_	10	~4.5%	< 1	RT	[110]
	TiO_2_	Gr	NH_3_	50	102.2%	36 ~ 16	25	[113]
			CH_3_OH	50	~11			[113]
			CO	50	~5			[113]
			H_2_S	50	~4			[113]
	Au	nitrogen doped Gr	C_6_H_6_O_2_	0.0016 μM	3			[111]
	Cu2+	SnO_2_	H_2_S	50	~89		RT	[108]
	Cu_3_(BTC)_2_(H_2_O)_3_	rGO	NH_3_	50	14.3%	13 ~ 22	RT	[112]
PANI	ZnO	SnO_2_	TEA	100	69		21	[118]
	ZnO	GO	NH_3_	50	38.31%	< 30		[114]
	Au	TiO_2_	NH_3_	10	48.6%	52 ~ 122	RT	[107]
			NH_3_	50	123%			[107]
			NO_2_	50	~28%			[107]
			CO	50	~19%			[107]
			H_2_S	50	~16%			[107]
			H_2_	50	~7%			[107]
			HCHO	50	~5%			[107]
			SO_2_	50	~3%			[107]
			O_3_	50	~2.5%			[107]
	SnO_2_	rGO	NH_3_	10	0.83	~80	RT	[115]
			LPG	10	~0.23			[115]
			CO_2_	10	~0.46			[115]
			C_2_H_6_O	10	~0.17			[115]
	Au	In_2_O_3_	NH_3_	100	~46	118 ~ 144	RT	[109]
			NH_3_	10	~9.5			[109]
			NO	10	~1			[109]
			CH_2_O	10	~1			[109]
			C_6_H_6_	10	~1			[109]
			C_7_H_8_	10	~1			[109]
			CH_3_COCH_3_	10	~0.9			[109]
			C_2_H_4_	100	~0.9			[109]
			CH_4_	100	~0.9			[109]
	MoS_2_	MWCNT	NH_3_	0.25	11%	32 ~ 36	RT	[120]
			NH_3_	6	40%			[120]
			C_2_H_5_OH	6	~13%			[120]
			C_6_H_6_	6	~5%			[120]
			CH_4_	6	~2.5%			[120]
			CH_3_COCH_3_	6	~7%			[120]
			C_3_H_8_	6	~2.5%			[120]
	SnO_2_	rGO	H_2_S	0.2	23.9%	82 ~ 78	25	[116]
	ZnO	S and N co-doped GQDs	CH_3_COCH_3_	0.5	2%	15 ~ 27	25	[117]
PANI	CuO+TiO_2_+ SiO_2_		NH_3_	100	45.67		RT	[119]

## 4. Conclusions and outlook

Outstanding achievements have been achieved in conducting polymer-based gas sensors in the past decades of years. However, to realize future commercialization, future efforts to increase the gas-sensing performance including high sensitivity, long-term stability, high selectivity, and high reversibility are still necessary. To date, in order to improve gas-sensing responses, a great number of doping inorganic nanomaterials and doping techniques has been developed to construct P-N or Schottky heterojunctions, improve electrical conductivity and modulate film morphology. In this review, we summarized recent advances in conducting polymer-inorganic nanocomposite-based gas sensors with excellent gas-sensing performances. Up to now, inorganic nanomaterials including metal oxides, metal, carbon nanotube, graphene, and binary nanocomposites (such as metal/metal oxide, metal oxide/carbon) to form conducting polymer-inorganic nanocomposites have been reported, which have been found to be an excellent platform for high-performance room-temperature gas sensing. This review clearly demonstrates that the sensing characteristics of the nanocomposite depend both on the composition and structural characteristics of individual constituents and the synergistic effects between them.

Although conducting polymer-inorganic nanocomposites have been adopted as excellent room-temperature gas-sensing elements in the literature, there are still more challenges to the understanding of the nature behind and further sensing performance enhancement toward practical applications. First, advanced analytical methods and theoretical modeling are necessary to analyze the intrinsic functions of inorganic nanomaterials responsible for gas-sensing enhancement effects, which could guide us to explore new nanocomposite systems toward practical applications. Second, from the point of view of synthesis methods, inorganic nanomaterials tend to aggregate in conducting polymer precursors and solvents, and nanocomposite films should be adhered well to substrates and electrodes. Exploring new synthesis methods of inorganic nanomaterials and nanocomposite films including modifying the surface of inorganic nanomaterials, adopting high-aspect-ratio inorganic nanomaterials, substrate surface functional group modification, etc., should be the next direction for researchers. Third, selectivity is still a major concern for practical applications of conducting polymer-inorganic nanocomposites. The development of target-analyte-specific nanocomposites with high sensitivity and long-term stability is urgently required. The interfaces between organic host materials and inorganic guest nanomaterials are the key toward high selectivity and sensitivity. Therefore, the interface needs to be more clearly clarified and understood by means of advanced interface analyze techniques. Forth, the robustness to the humidity of the gas sensor is important for practical applications because the environment is always complex with high humidity. Interestingly, PANI exhibits better sensing response and recovery properties at higher humidity, which makes PANI-based nanocomposites promising for high-humidity application cases. The sensing response enhancement is always contributed to the protonation doping effects of PANI by water molecules. However, the inherent mechanisms need to be systematically investigated, which will promote the applications of polymer-inorganic nanocomposites based gas sensor in practical high-humidity environments. We hope that this review will inspire the creation of new nanocomposite systems, synthesis methods, sensing mechanisms, and interface physics, and promote further development of high-performance room-temperature gas sensors.
